# TRPV1 Inhibits the Ventilatory Response to Hypoxia in Adult Rats, but Not the CO_2_-Drive to Breathe

**DOI:** 10.3390/ph12010019

**Published:** 2019-01-24

**Authors:** Luis Gustavo A. Patrone, Jaime B. Duarte, Kênia Cardoso Bícego, Alexandre A. Steiner, Andrej A. Romanovsky, Luciane H. Gargaglioni

**Affiliations:** 1 Department of Animal Morphology and Physiology, Faculty of Agricultural and Veterinarian Sciences, UNESP at Jaboticabal, Rod. Prof. Paulo Donato Castellane s/n, Jaboticabal SP 14870-000, Brazil; luispatrone@hotmail.com (L.G.A.P.); jaime_bizarri_duarte@hotmail.com (J.B.D.); keniacb@yahoo.com.br (K.C.B.); 2 Department of Immunology, Institute of Biomedical Sciences, University of Sao Paulo, Sao Paulo 05508-090, Brazil; steiner.temperature@gmail.com; 3 Thermoregulation and Systemic Inflammation Laboratory (FeverLab), Trauma Research, St. Joseph’s Hospital and Medical Center, Phoenix, AZ 85013, USA; Andrej.Romanovsky@dignityhealth.org

**Keywords:** ventilation, hypercapnia, channels, chemosensitivity, hypothermia, blood pressure

## Abstract

Receptors of the transient receptor potential (TRP) channels superfamily are expressed in many tissues and have different physiological functions. However, there are few studies investigating the role of these channels in cardiorespiratory control in mammals. We assessed the role of central and peripheral TRPV1 receptors in the cardiorespiratory responses to hypoxia (10% O_2_) and hypercapnia (7% CO_2_) by measuring pulmonary ventilation (${\dot{V}}_{E}$), heart rate (HR), mean arterial pressure (MAP) and body temperature (Tb) of male Wistar rats before and after intraperitoneal (AMG9810 [2.85 µg/kg, 1 mL/kg]) or intracebroventricular (AMG9810 [2.85 µg/kg, 1 µL] or AMG7905 [28.5 μg/kg, 1 µL]) injections of TRPV1 antagonists. Central or peripheral injection of TRPV1 antagonists did not change cardiorespiratory parameters or Tb during room air and hypercapnic conditions. However, the hypoxic ventilatory response was exaggerated by both central and peripheral injection of AMG9810. In addition, the peripheral antagonist blunted the drop in Tb induced by hypoxia. Therefore, the current data provide evidence that TRPV1 channels exert an inhibitory modulation on the hypoxic drive to breathe and stimulate the Tb reduction during hypoxia.

## 1. Introduction

Breathing is a vital behavior that is generated by a medullary region, called the preBötzinger Complex (preBötC), and is regulated by a complex system that depends on chemical feedback of the carotid body (peripheral chemoreceptors) and central chemoreceptors [1,2,3]. Hypoxia and hypercapnia are potent chemical stimuli to the respiratory system, which, in turn, is modulated by many neurotransmitters and receptors, including the transient receptor potential (TRP) channels [4,5].

The TRP superfamily consists of a large number of nonselective cation channels that are activated by several stimuli, including heat, acid, pressure, shear stress, mechanical stretch, oxidative stress, phospholipids, etc. [4,6,7,8]. TRPC1/3/4/5 and 6 channels are expressed in the carotid chemosensory pathway [9], and also contribute to acid sensing in a respiratory chemosensitive nucleus [4], suggesting that these channels can be involved in the ventilatory response to hypoxia and hypercapnia. Of interest here are the vanilloid-1 (TRPV1) channels, which are capsaicin-sensitive, heat-sensitive, and proton-activated calcium channels that have a significant role in thermoregulation in response to changes in environmental and body temperatures [10,11,12]. TRPV1 receptors are widely expressed in peripheral and central locations, including the dorsal horn of the spinal cord [13], the brainstem and forebrain [14,15,16,17,18]. The expression of these receptors in regions such as the preBötC, parafacial respiratory region, locus coeruleus, nucleus of solitary tract [19,20,21], and also in petrosal neurons innervating carotid body glomus cells [22], suggests a role in the control of breathing and chemoreception.

Supporting the role of TRPV1 channels in respiratory modulation, bath application of capsaicin (TRPV1 agonist) in the medulla of a brainstem-spinal cord preparation has many effects on the respiratory center, including potent (but transient) inhibitory and subsequent excitatory effects on the respiratory rhythm and the periodical augmentation of the inspiratory burst pattern [20]. More recently, Barrett et al. [23] demonstrated that peripheral activation of TRPV1 receptors by piperine in neonatal rats prior to heat exposure enhances the ventilatory response to hyperthermia, which is attenuated by the TRPV1 antagonist, AMG9810. Nevertheless, no studies have assessed the participation of these channels in breathing control in unanesthetized adult rats.

Emerging evidence also supports the role of the TRPV1 as an important regulator of cardiovascular function, having a protective role against cardiovascular injury [24]. In agreement, Sun et al. [25] showed that TRPV1 is expressed along the entire baroreceptive afferent pathway. Blockade of TRPV1 receptors significantly blunts the baroreflex and decreases the maximum gain of baroreflex function in the high blood pressure range, indicating that TRPV1-expressing baroreceptive afferents are important for the feedback control of blood pressure stability [25].

TRPV1 antagonists have been considered a novel tool for pharmacological manipulation of pain. AMG9810 is a selective and competitive TRPV1 antagonist of capsaicin activation and blocks all known modes of TRPV1 activation, including protons, heat and endogenous ligands, anandamide, N-arachidonyl dopamine and oleoyldopamine [26], whereas AMG7905 is a potent blocker of the capsaicin mode and potent potentiator of the proton mode of TRPV1 activation [27,28], and possibly of the heat mode [27]. However, as side effect these drugs can cause hyperthermia associated with increased thermogenesis and skin vasoconstriction [10,29,30,31] or, in some cases, hypothermia [28]. Nevertheless, it is still unclear if the TRPV1 channels have effects on cardiorespiratory control in basal, hypoxic and hypercapnic conditions. Therefore, in the present study, we used a pharmacological approach, where intracerebral and peripheral injections of the TRPV1 antagonists were used to investigate the role of TRPV1 in cardiorespiratory control in unanesthetized adult rats.

## 2. Results

### 2.1. Effect of icv Injection of AMG9810 and AMG7905 on ${\dot{V}}_{E}$, MAP, HR and Tb in Normocapnic and Hypercapnic Conditions

**Figure 1:**
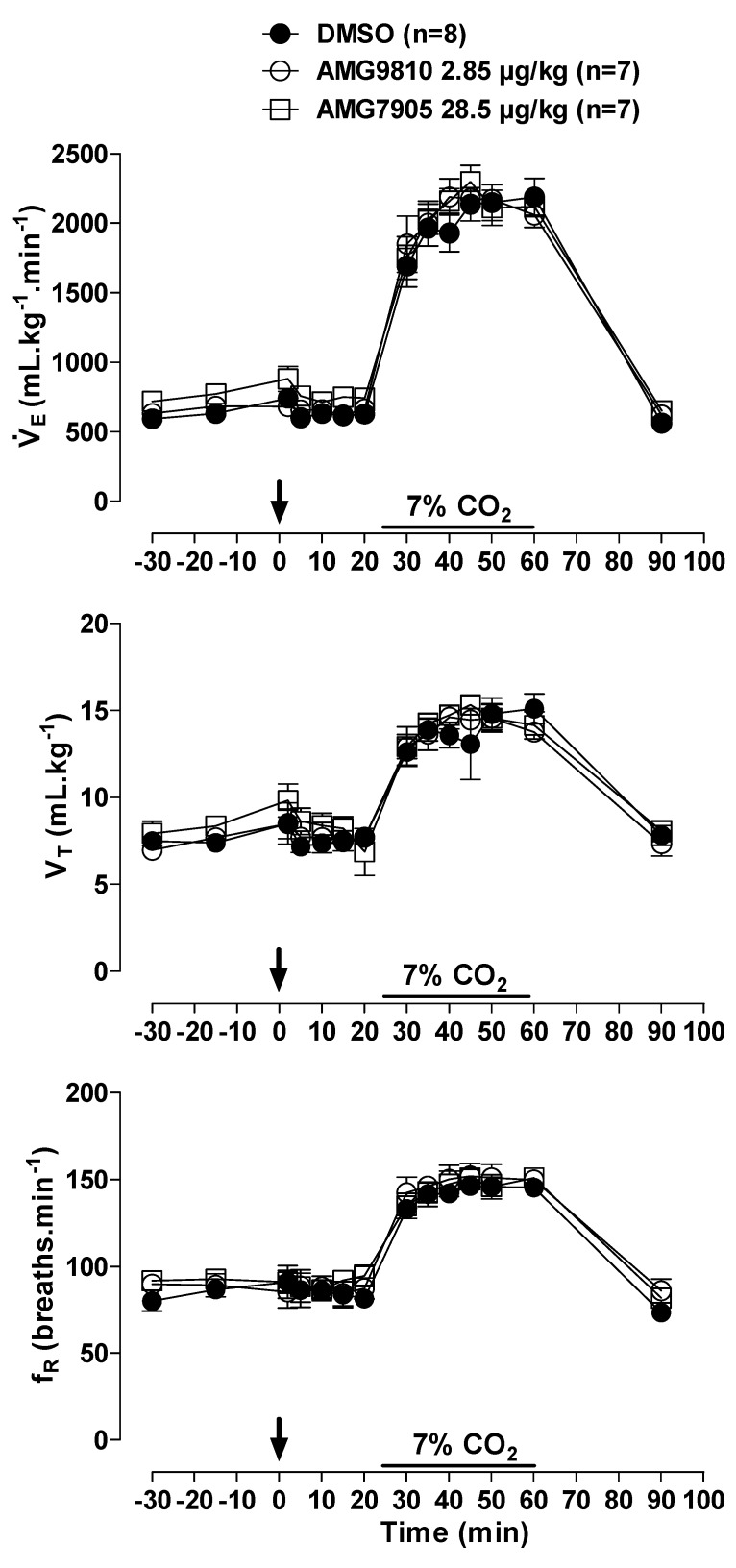
Effects of intracerebroventricular (icv) microinjection of vehicle (DMSO), AMG9810 (TRPV1 antagonist—2.85 µg/kg, 1 µL) and AMG7905 (TRPV1 antagonist—28.5 µg/kg, 1 µL) on ventilation (${\dot{V}}_{E}$), tidal volume (V_T_) and respiratory frequency (fR) of rats during normocapnia and hypercapnia (7% CO_2_). The arrow indicates the time of the microinjection. The hypercapnia duration is represented by a horizontal line on the graph. Values are expressed as mean ± S.E.M.

**Figure 2:**
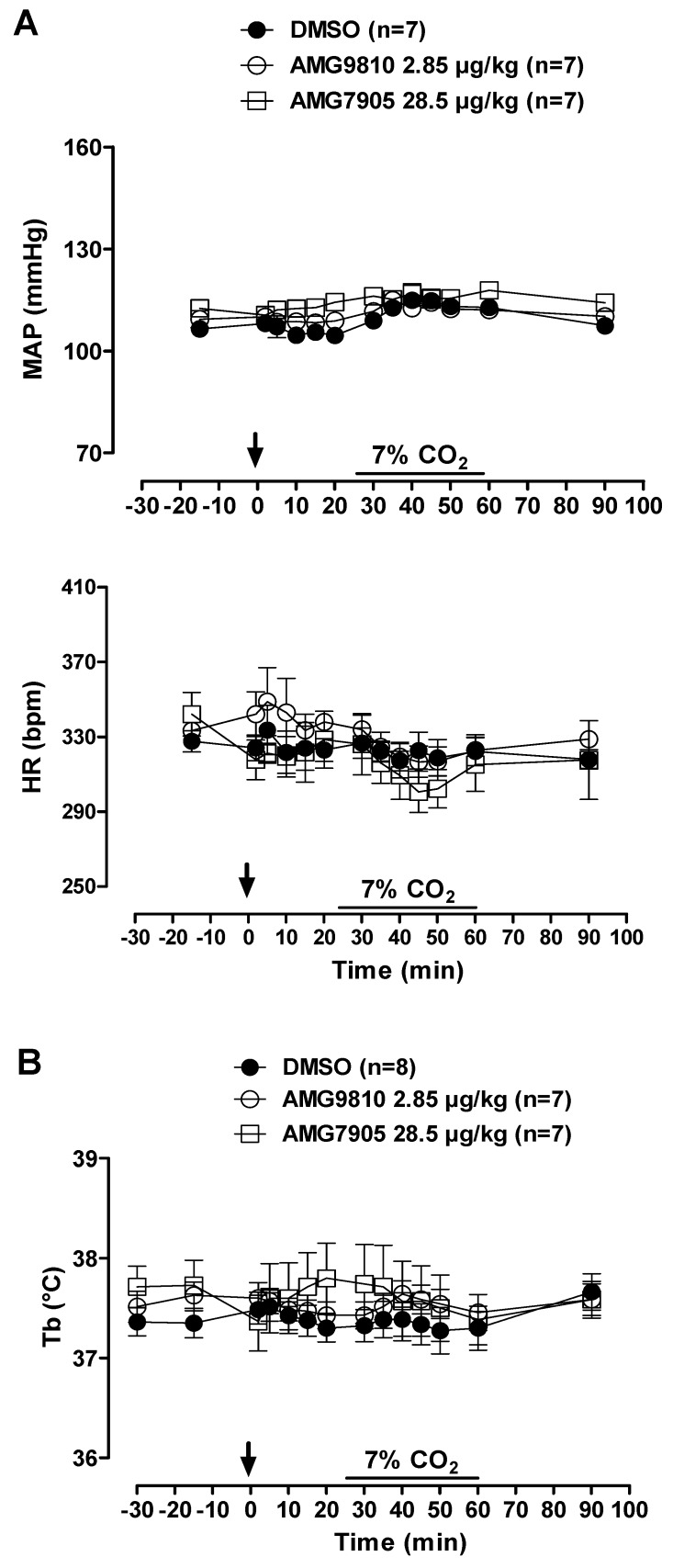
Effects of intracerebroventricular (icv) microinjection of vehicle (DMSO), AMG9810 (TRPV1 antagonist—2.85 µg/kg, 1 µL) and AMG7905 (TRPV1 antagonist—28.5 µg/kg, 1 µL) on (**A**) mean arterial pressure (MAP) and heart rate (HR) and (**B**) body temperature (Tb) of rats during normocapnia and hypercapnia (7% CO_2_). The arrow indicates the time of the microinjection. The hypercapnia duration is represented by a horizontal line on the graph. Values are expressed as mean ± S.E.M.

In room air conditions, microinjection of AMG9810 and AMG7905 had no effect on, MAP or HR (Figure 1 and Figure 2A). Hypercapnia induced a progressive increase in ventilation in all animals (effect of time: *p* < 0.001; Figure 1), with no difference among groups.

MAP and HR were not affected by microinjection of TRPV1 antagonists in hypercapnic conditions. In addition, hypercapnia did not influence cardiovascular variables (Figure 2A).

Under normocapnia and hypercapnia, neither vehicle nor AMG9810 or AMG7905 microinjection affected Tb (Figure 2B).

### 2.2. Effect of icv Injection of AMG9810 and AMG7905 on ${\dot{V}}_{E}$, MAP, HR and Tb in Normoxic and Hypoxic Conditions

**Figure 3:**
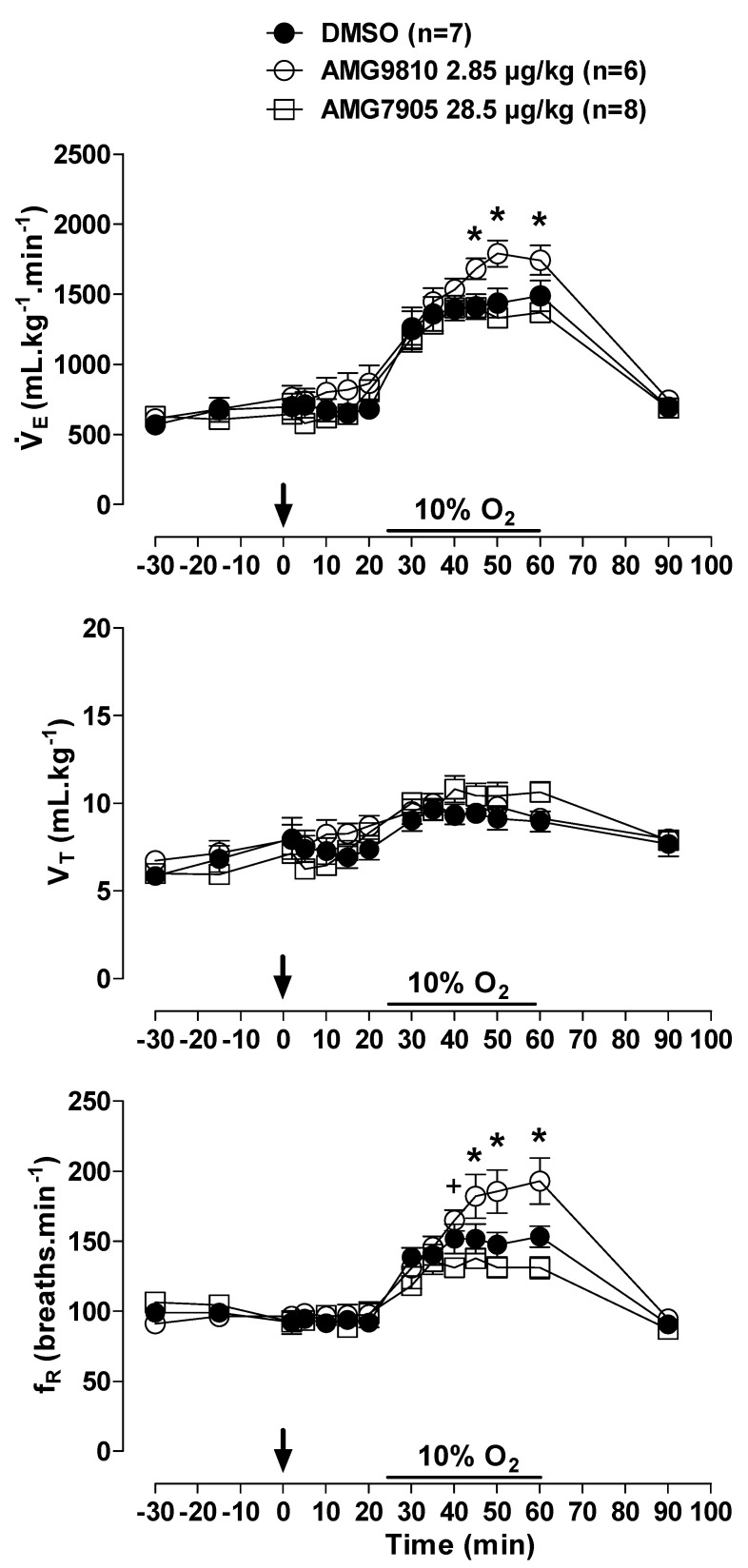
Effects of intracerebroventricular (icv) microinjection of vehicle (DMSO), AMG9810 (TRPV1 antagonist—2.85 µg/kg, 1 µL) and AMG7905 (TRPV1 antagonist—28.5 µg/kg, 1 µL) on ventilation (${\dot{V}}_{E}$), tidal volume (V_T_) and respiratory frequency (fR) of rats during normoxia and hypoxia (10% O_2_). The arrow indicates the time of the microinjection. The hypoxia duration is represented by a horizontal line on the graph. Values are expressed as mean ± S.E.M. * Significant differences between AMG9810 with vehicle and AMG7905 groups. ^+^Significant difference between AMG7905 and AMG9810 groups.

Hypoxia caused an increase in in all animals (effect of time: *p* < 0.001; Figure 3); however, the hypoxic ventilatory response was significantly more pronounced in the animals that received central injection of AMG9810 at 45, 50 and 60 min of hypoxic exposure (effect of treatment: *p* < 0.05; Figure 3), due to a higher fR (effect of treatment: *p* < 0.01; Figure 3). Central AMG7905 did not change the hypoxic ventilatory response. 

**Figure 4:**
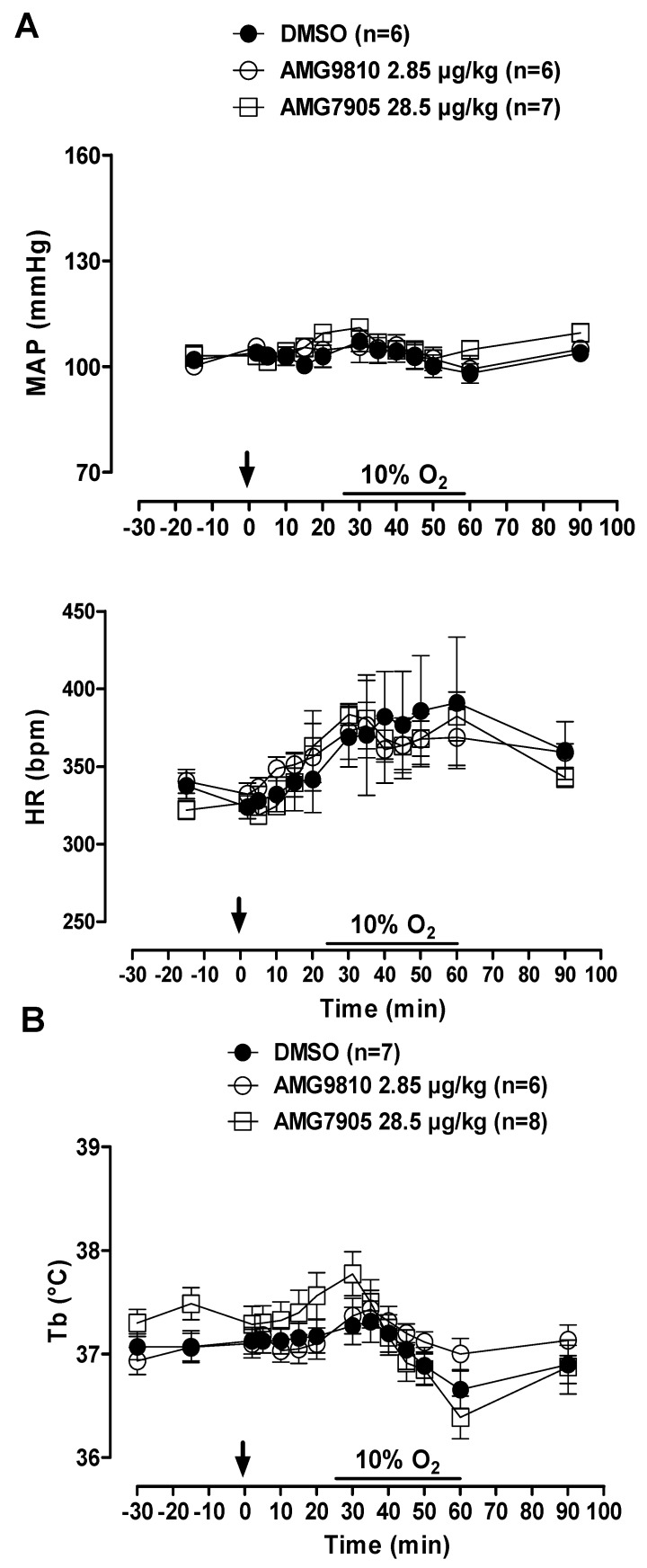
Effects of intracerebroventricular (icv) microinjection of vehicle (DMSO), AMG9810 (TRPV1 antagonist—2.85 µg/kg, 1 µL) and AMG7905 (TRPV1 antagonist—28.5 µg/kg, 1 µL) on (**A**) mean arterial pressure (MAP) and heart rate (HR) and (**B**) body temperature (Tb) of rats during normoxia and hypoxia (10% O_2_). The arrow indicates the time of the microinjection. The hypoxia duration is represented by a horizontal line on the graph. Values are expressed as mean ± S.E.M.

MAP and HR were not affected by microinjection of TRPV1 antagonists in hypoxic conditions. In addition, hypoxia did not significantly influence cardiovascular variables (Figure 4A). Hypoxia caused a similar decrease in Tb in all groups (*p* < 0.001; Figure 4B).

### 2.3. Effect of Intraperitoneal Injection of AMG9810 on ${\dot{V}}_{E}$, MAP, HR and Tb in Normocapnic and Hypercapnic Conditions

**Figure 5:**
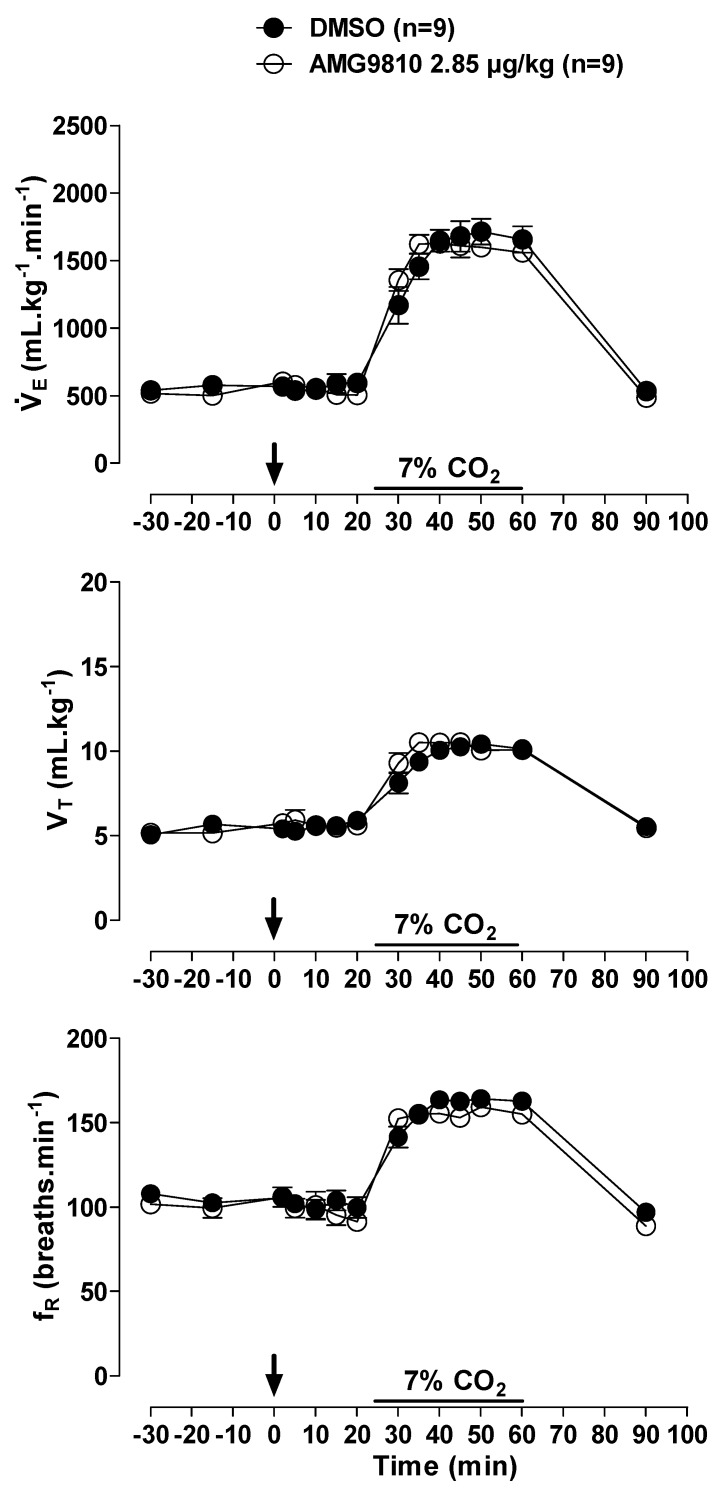
Effects of intraperitoneal (ip) injection of vehicle (DMSO) and AMG9810 (TRPV1 antagonist—2.85 µg/kg, 1 mL/kg) on ventilation (${\dot{V}}_{E}$), tidal volume (V_T_) and respiratory frequency (fR) of rats during normocapnia and hypercapnia (7% CO_2_). The arrow indicates the time of the microinjection. The hypercapnia duration is represented by a horizontal line on the graph. Values are expressed as mean ± S.E.M.

**Figure 6:**
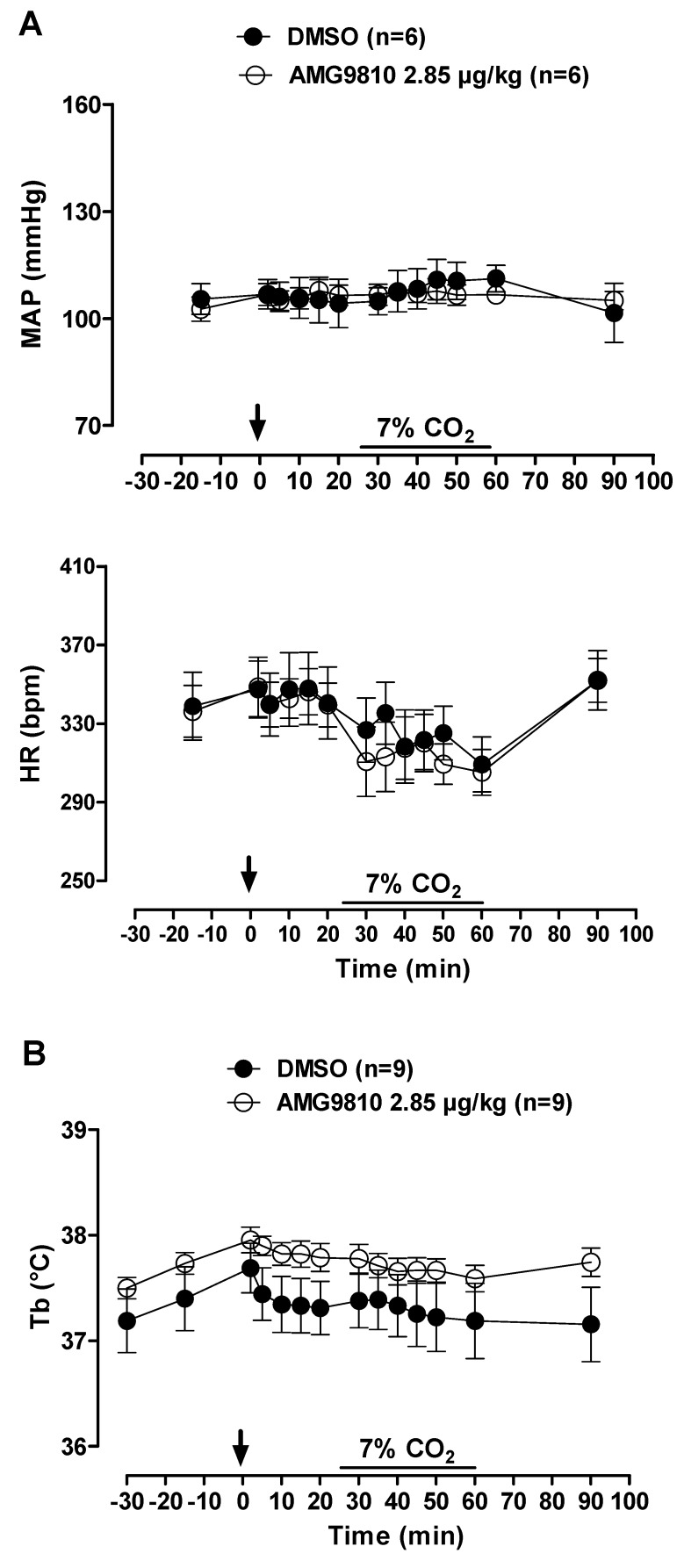
Effects of intraperitoneal (ip) injection of vehicle (DMSO) and AMG9810 (TRPV1 antagonist—2.85 µg/kg, 1 mL/kg) on (**A**) mean arterial pressure (MAP) and heart rate (HR) and (**B**) body temperature (Tb) of rats during normocapnia and hypercapnia (7% CO_2_). The arrow indicates the time of the microinjection. The hypercapnia duration is represented by a horizontal line on the graph. Values are expressed as mean ± S.E.M.

During normocapnia, intraperitoneal injection of AMG9810 did not affect ${\dot{V}}_{E}$, MAP, HR or Tb (Figure 5 and Figure 6A,B). Hypercapnia caused an increase in ventilation in all groups (effect of time: *p* < 0.001; Figure 5), with no difference between groups. Hypercapnia did not change MAP, HR or Tb, and intraperitoneal injection of a TRPV1 antagonist did not significantly affect cardiovascular variables or Tb in hypercapnic conditions (Figure 6A,B).

### 2.4. Effect of Intraperitoneal Injection of AMG9810 on ${\dot{V}}_{E}$, MAP, HR and Tb in Normoxic and Hypoxic Conditions

**Figure 7:**
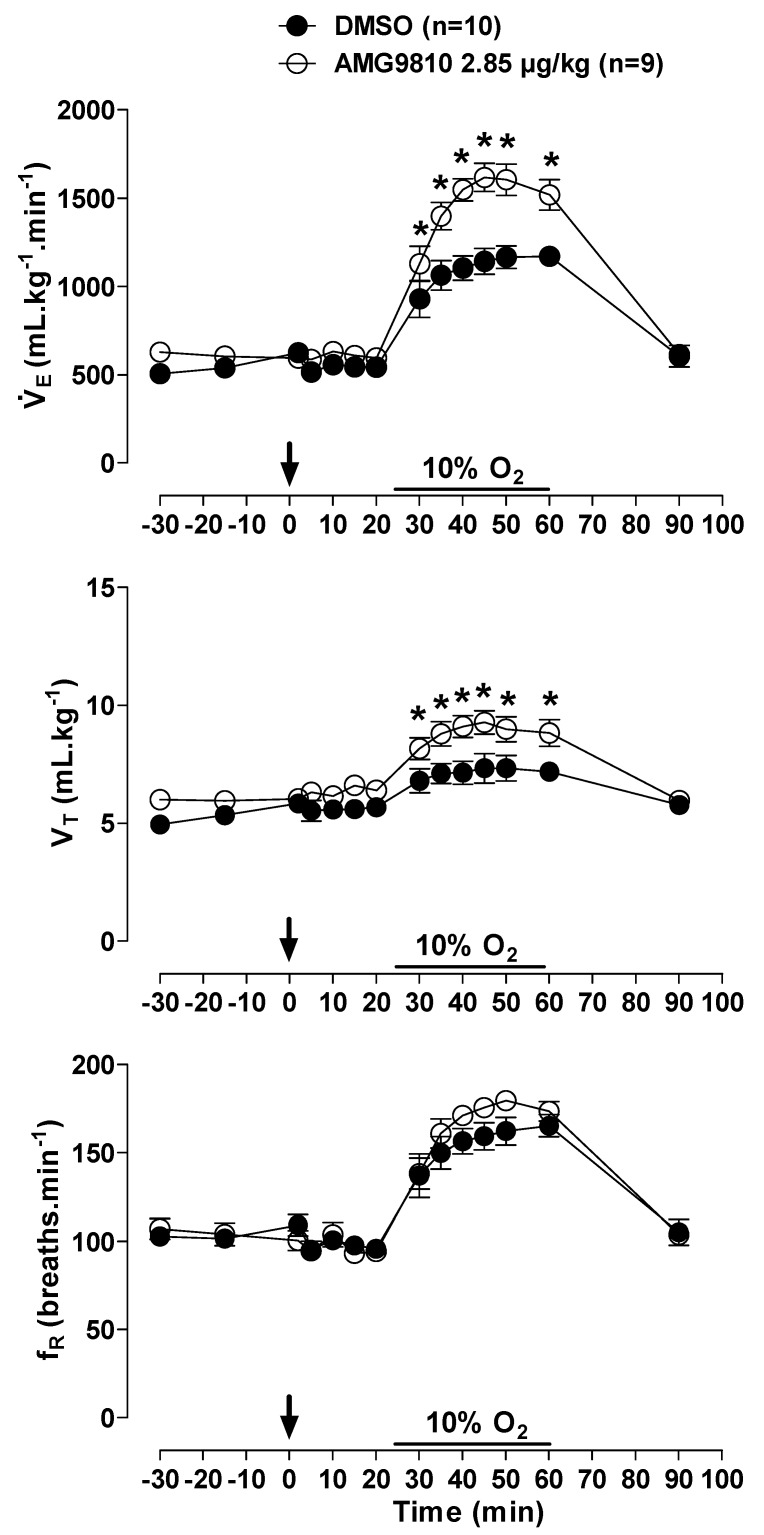
Effects of intraperitoneal (ip) injection of vehicle (DMSO) and AMG9810 (TRPV1 antagonist—2.85 µg/kg, 1 mL/kg) on ventilation (${\dot{V}}_{E}$), tidal volume (V_T_) and respiratory frequency (fR) of rats during normoxia and hypoxia (10% O_2_). The arrow indicates the time of the microinjection. The hypoxia duration is represented by a horizontal line on the graph. Values are expressed as mean ± S.E.M. * Significant difference between vehicle and AMG9810 groups.

Hypoxia caused an increase in in all animals (effect of time: *p* < 0.001; Figure 7); however, the hypoxic ventilatory response was significantly more pronounced in the animals that received peripheral injection of AMG9810 at 30, 35, 40, 45, 50, and 60 min of hypoxic exposure (effect of treatment: *p* < 0.02; Figure 7), due to a higher V_T_ (effect of treatment: *p* < 0.03; Figure 7). 

**Figure 8:**
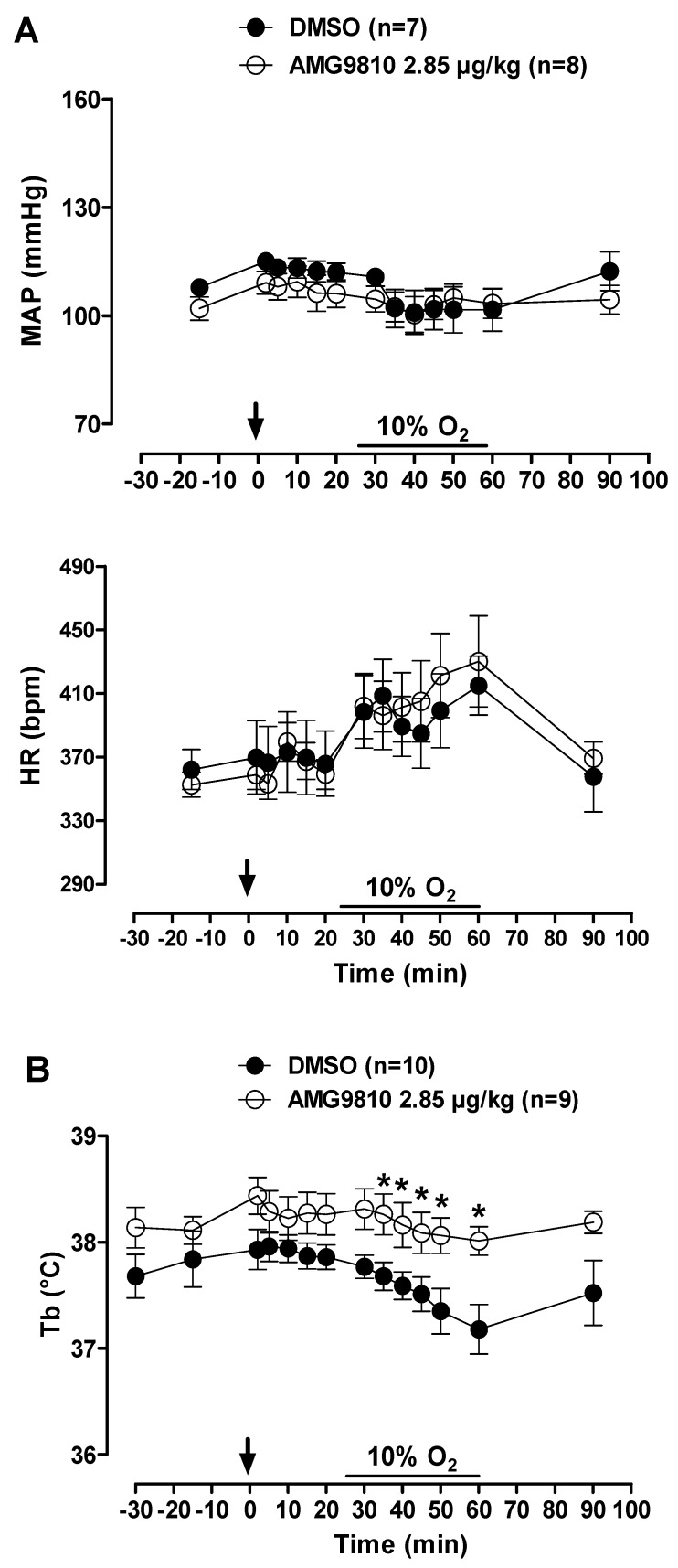
Effects of intraperitoneal (ip) injection of vehicle (DMSO) and AMG9810 (TRPV1 antagonist—2.85 µg/kg, 1 mL/kg) on (**A**) mean arterial pressure (MAP) and heart rate (HR) and (**B**) body temperature (Tb) of rats during normoxia and hypoxia (10% O_2_). The arrow indicates the time of the microinjection. The hypoxia duration is represented by a horizontal line on the graph. Values are expressed as mean ± S.E.M. * Significant difference between vehicle and AMG9810 groups.

MAP and HR were not affected by peripheral injection of a TRPV1 antagonist in hypoxic conditions. Additionally, hypoxia did not alter the cardiovascular variables (Figure 8A).

**Figure 9:**
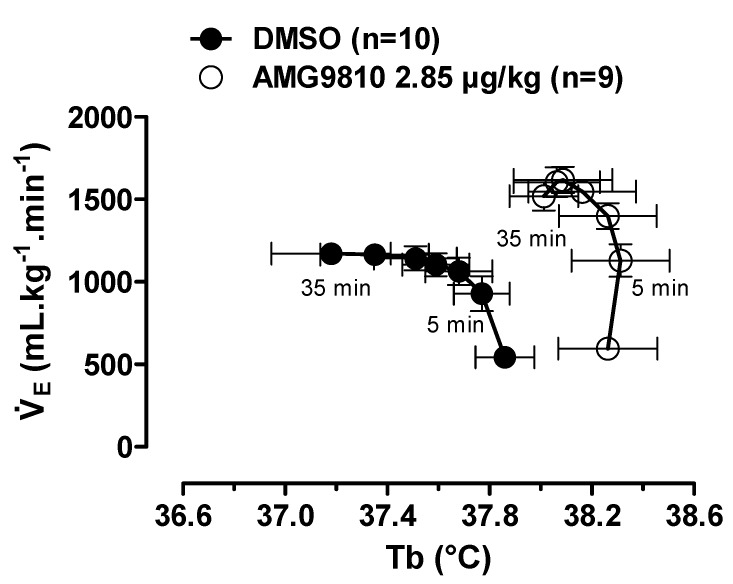
Relationship between pulmonary ventilation (${\dot{V}}_{E}$; data from Figure 7) and body temperature (Tb; data from Figure 8B) in rats treated with vehicle (DMSO) or AMG9810 (TRPV1 antagonist—2.85 µg/kg, 1 mL/kg). The direction of time is shown with arrows, and the times corresponding to the earliest (5 min) and latest (35 min) hypoxia exposure data points are indicated. Values are expressed as mean ± S.E.M.

Hypoxia caused a decrease in Tb (effect of time: *p* < 0.001) that was attenuated by intraperitoneal injection of AMG9810 at 35, 40, 45, 50 and 60 min of hypoxic exposure (effect of treatment: *p* < 0.04; Figure 8B). Additionally, these effects of TRPV1 antagonism may be better evidenced in Figure 9, in which the values of ${\dot{V}}_{E}$ by Tb are plotted under normoxic (20 min after ip injection, 5 min before hypoxia) and hypoxic (5–35 min of hypoxia) conditions. It is possible to observe an increase in ventilation, which achieves a plateau, while Tb continues to decrease in the vehicle group. On the other hand, in the AMG9810 group, ventilation increases more, achieving higher levels without any further decrease in Tb. 

### 2.5. Effect of icv and Intraperitoneal Injection of AMG9810 on Blood Gases and pH in Normoxic, Normocapnic, Hypercapnic and Hypoxic Conditions

Exposure of vehicle- and TRPV1 antagonist-injected groups to 7% CO_2_ caused a similar significant increase in *P*aCO_2_ (*p* < 0.001) and *P*aO_2_ (*p* < 0.001) and a decrease in pHa (*p* < 0.001) (Table 1A). None of the experimental conditions had any significant effect on plasma HCO_3_^–^ (Table 1A). 

*P*aO_2_, *P*aCO_2_ and HCO_3_^–^ decreased (*p* < 0.05) and arterial pH increased (*p* < 0.05) during hypoxia in all groups, compared to normoxic conditions (Table 1B and Table 2). Peripheral or central TRPV1 antagonist injection did not change blood gases, pH or HCO_3_^–^.

## 3. Discussion

Several studies have reported the role of TRPV1 on thermoregulation and nociception; however, none has addressed the influence of this channel on ventilatory and cardiovascular responses to hypoxia and hypercapnia in unanesthetized rats. Our results indicate that central and peripheral TRPV1 receptors play an important role in regulating breathing and body temperature during hypoxia, since antagonism with AMG9810 caused a significant increase in ventilation and attenuation of Tb reduction under hypoxic exposure. Nevertheless, we found that TRPV1 receptors do not participate in cardiorespiratory control during room air and hypercapnic conditions.

In the current study, both central and peripheral administration of AMG9810 or central AMG7905 did not change ventilation or cardiovascular parameters during room air conditions, suggesting that TRPV1 receptors may have no tonic role in cardiorespiratory control. The TRPV1 channel is a polymodal signal detector that can be activated by heat, protons, and chemical ligands, such as capsaicin [27,31,32,33]. In fact, capsaicin acting on the preBötC causes release of glutamate and depletion of substance P, interrupting the respiratory rhythm in neonatal rat slices [34]. More recently, Tani et al. [20], using brainstem-spinal cord preparations from newborn rats, demonstrated that capsaicin caused a transient decrease, followed by an increase, in respiratory nerve activity. The excitatory phase was partially blocked by AMG9810 (10 μM), whereas the initial inhibitory effect was not suppressed by the TRPV1 antagonist. The authors found a cessation of breathing after capsaicin application only when the parafacial nucleus was not present in the preparation. Our study is the first to demonstrate in unanesthetized adult rats that the TRPV1 channel may not play a role in breathing regulation during basal conditions.

In the current study, hypercapnia promoted an increase in ventilation (Figure 1) and respiratory acidosis (Table 1A). As TRPV1 channels have H^+^ sensitivity [31,35], administration of a TRPV1 antagonist would result in a reduced hypercapnic ventilatory response. However, no difference in CO_2_ ventilatory response was observed among groups, suggesting a lack of TRPV1 involvement in the CO_2_-drive to breathe, at least at this level of respiratory acidosis (pH ~ 7.3). Indeed, previous studies have demonstrated that TRPV1 is activated by pH ≤ 6 [35,36,37]. It is possible that other channels are involved in the modulation of the central chemoreflex, at least in a more physiological pH range. In fact, TRPC4 and TRPC5 are activated at pH 7.5–6.5 [38,39], and TRPC5 mediates the stimulatory effect of hypercapnia on locus coeruleus neurons, a central CO_2_/pH chemosensitive area [4]. 

Regarding the hypoxic challenge, 10% inspired O_2_ promoted an increase in ventilation in the vehicle-, AMG9810- and AMG7905-treated groups as a result of an increase in V_T_ and fR. Central and peripheral injection of AMG9810 increased the hypoxic ventilatory response, compared to the vehicle- and AMG7905-treated groups. Since AMG9810 injection increased ventilation only during hypoxic exposure and not during normoxia or hypercapnia, our data suggest that central and peripheral TRPV1 channels exert a specific inhibitory modulation on the ventilatory response to lower O_2_ situations. Interestingly, previous studies have demonstrated an attenuated hypoxic ventilatory in adult rats treated with 50 mg/kg of capsaicin neonatally [40,41]. According to the authors, the blunted hypoxic response in the capsaicin-treated rats could be due to either degeneration of unmyelinated carotid body afferents and/or depletion of substance P from the carotid body. However, a more recent study has demonstrated that central and peripheral chemoreceptors chemosensitivity was not decreased by neonatal capsaicin treatment (25 mg/kg) in lambs [42]. According to the authors, the differences observed between the studies might be due to the fact that unmyelinated fibers and SP are less important within the afferent pathway leading from carotid body to respiratory neurons in sheep than in rodents. 

In the current study, the enhanced hypoxic ventilatory response was only observed with AMG9810, and not with AMG7905, injection. Since AMG7905 is less specific and do not block endogenous ligands of cannabinoid on TRPV1 receptors, the absence of response with the central injection of AMG7905 suggest that the results obtained with the antagonist AMG9810 during hypoxia is not mediated by cannabinoid ligands. Therefore, it is possible that the activation of all modes of the TRPV1 channel are needed to inhibit the hypoxic drive to breath, as demonstrated also by the action of AMG9810 as an antihyperalgesic drug [26]. Since both intraperitoneal and icv application of AMG9810 promoted 41% and 24% increases in the ventilatory response to hypoxia, respectively, it is difficult to distinguish whether the effect is central or peripheral. In this context, Gavva et al. [43] reported that small amounts of even some peripherally restricted TRPV1 antagonists do cross the blood brain barrier; therefore, we cannot completely rule out that peripherally-injected AMG9810 is acting centrally. In this regard, it is well known that TRPV1 receptors are present in brainstem medullary neurons, and also the carotid body [20,22], and both sites can contribute to the enhanced hypoxic ventilatory response. Interestingly, central AMG9810 potentiates the hypoxic drive to breathe, mainly acting on respiratory frequency, whereas peripheral AMG9810 increased tidal volume. These differences may be related to the fact that peripheral AMG9810 is acting mainly on the carotid body, whereas central AMG9810 is acting on brainstem areas that control breathing frequency, suggesting peripheral and central actions of TRPV1 channels on breathing control during low O_2_ conditions. In agreement with this hypothesis, Smith and Mills [44] suggested that the carotid body mostly drives V_T_; therefore, peripheral TRPV1 antagonism is possibly increasing the sensitivity of peripheral chemoreceptors. Previously, Roy et al. [22] have demonstrated that TRPV1 receptors in the carotid body chemosensory afferents are mainly responsible for the anandamide and heat sensitivity of the carotid body. 

Regarding Tb, TRPV1 antagonists can cause hyper or hypothermia, depending on the activation mode upon which they act. As previously cited, AMG9810 is a polymodal TRPV1 antagonist that blocks all three modes of TRPV1 activation (proton, heat and capsaicin) [28] and causes hyperthermia by disinhibiting thermogenesis and skin vasoconstriction. Previous studies suggested that the ability of TRPV1 antagonists to cause hyperthermia relays to their potency to block TRPV1 activation by protons [33], especially within the abdominal cavity [29]. In contrast, since the hyperthermic effect is due to blockade of the proton mode, AMG7905 normally causes hypothermia because of its potentiation of the proton mode [28]. In the current study, none of the antagonists affected Tb in basal conditions. Regarding AMG7905, the lack of effect must be related to the site of injection (central), since this antagonist acts mainly in the abdominal cavity [28] to cause hypothermia. As for AMG9810, the dose used peripherally could be too low to cause hyperthermia (2.85 µg/kg) compared to other studies [28,33]. Notwithstanding, this dose was sufficient to attenuate the drop in Tb caused by hypoxia, similar to how too low doses of TRPV1 antagonists attenuate or prevent anesthesia-associated hypothermia [45]. Considering a CSF volume of 90 μL in an adult rat [46] and that 3 mM of AMG9810 in the current study is totally diluted in this volume, the final concentration would be 0.032 mM, which is 405 times the IC_50_ for in vitro capsaicin inhibition (79 × 10^−6^ mM) [10]. If the same calculation is applied for AMG7905 (20 mM), the final CSF concentration would be 0.22 mM, which is 5.600 times higher than the IC_50_ for in vitro capsaicin inhibition [27]. The possibility of total dilution in CSF could be happened, considering a CSF flow is 2 µL/min [47] and that the ventilatory responses to hypoxia were only observed 40 min after drug injection.

It is well established that hypoxia reduces Tb by reducing heat production—which involves a decreasing of the hypothalamic thermogenic threshold [48]—and increasing heat loss and cold-seeking behavior [49,50,51,52]. Such evidence supports the idea of the hypoxia-induced Tb drop as a regulated phenomenon [48,50,52,53,54,55]. The increase in hypoxic ventilatory response after peripheral application of AMG9810 was higher (41%) compared to central (24%) injection, which, at least in part, possibly involves a smaller reduction of Tb during a hypoxic challenge (Figure 9). Taken together our data indicate that the activation of TRPV1 receptors not only induces a drop in Tb, but also inhibits the energetically costly hyperventilation during hypoxia exposure, responses that are considered protective during low O_2_ situations [52,53,54,55].

In conclusion, the present results provide evidence that central and peripheral TRPV1 receptors play an important inhibitory modulation on breathing and body temperature during hypoxia, suggesting a protective role during low O_2_ situations, reducing energetically expensive responses, such as the hypoxic ventilatory response, but it does not play a role in cardiovascular regulation, at least with the doses used in the present study. 

## 4. Materials and Methods

### 4.1. Animals

Experiments were performed on unanesthetized adult male Wistar rats, weighing 300–350 g. The animals had free access to water and food, and were housed in a temperature-controlled chamber maintained at 24–26 °C (ALE 9902001; Alesco Ltd.a., Monte Mor, SP, Brazil) with a 12:12 h light:dark cycle (lights on at 6:30 a.m.). The study was conducted in compliance with the guidelines of the National Council for the Control of Animal Experimentation (CONCEA, MCT, Belo Horizonte Area, Brazil) and with the approval of the local Animal Care and Use Committee (CEUA, FACV-UNESP Jaboticabal; Protocol: 06585/14).

### 4.2. Surgeries and Microinjection

All surgical procedures were performed under anesthesia with 100 mg/kg of ketamine (Union National Pharmaceutical Chemistry S/A, Embu-Guaçu, SP, Brazil) and 10 mg/kg of xylazine (Laboratories Calier S/A, Barcelona, Spain), administered intraperitoneally (i.p.).

Seven days before the experiments, the head was shaved, and the skin was sterilized with betadine solution and alcohol. Then, a guide cannula (0.7 mm o.d. and 15 mm in length) was intracerebroventricularly (icv) implanted in the fourth ventricle (4V), using the following coordinates: 11.9 mm caudal from bregma, at the midline, and 7.4 mm ventral to the surface of the skull [56]. The displacement of the meniscus in a water manometer ensured correct positioning of the cannula in the 4V. The cannula was attached to the bone using stainless steel screws and acrylic cement. A tight-fitting stylet was kept inside the guide cannula to prevent occlusion. Postoperatively, animals were treated with antibiotic (enrofloxacin, 10 mg/kg, intramuscular) and analgesic (flunixin meglumine, 2.5 mg/kg, subcutaneous) agents. 

One day before the experiments, rats underwent two surgeries under tribromoethanol anesthesia administered intraperitoneally (10 mL/kg). First, a catheter [PE-10 connected to PE-50 (Clay Adams, Parsippany, NJ, USA)] was inserted into the abdominal aorta through the femoral artery to measure pulsatile arterial pressure and blood gases. The catheter was tunneled subcutaneously and exteriorized through the back of the neck. On the following day, this catheter was connected to the pressure transducer in a way that allowed free movement of the rat. The pulsatile arterial pressure was measured using a pressure transducer (TSD 104A, Biopac Systems, Santa Barbara, CA, USA) connected to an amplifier (DA 100C, Biopac Systems). Heart rate (HR) and mean arterial pressure (MAP) were quantified from the pulsatile arterial pressure records using the same system (MP100 ACE, Biopac Systems). For body temperature (Tb) measurements, a temperature datalogger (SubCue Data Loggers, Calgary, AB, Canada) was implanted in the abdominal cavity through a midline laparotomy. The datalogger was programmed to acquire data every 5 min. These temperature values were used to calculate pulmonary ventilation. 

A 5-μL Hamilton syringe and a dental injection needle (200 μm o.d., Mizzy, Reno, NV-USA) connected to a PE-10 tube was used to perform the microinjections into the 4V of unanesthetized rats. A volume of 1 µL of vehicle or drug solution was injected over a period of 20 s, and the needle was removed from the guide cannula after an additional 30 s to avoid reflux. All injections were performed using a microinjector machine (model 310, Stoelting CO., Wood Dale, IL, USA). For ip injections, the volume was established according to the animal’s body weight (1 mL/kg), and the injection was performed over a period of 15 s. 

### 4.3. Drugs and Gas Mixture

TRPV1 antagonists used, AMG9810 and AMG7905, were purchased from Enzo Life Sciences (Farmingdale, NY, USA) and Glixx Laboratories (Hopkinton, MA, USA), respectively.

AMG9810 (2.85 µg/kg) was applied centrally (1 µL) and intraperitoneally (1 mL/kg), and AMG7905 was only injected centrally (28.5 µg/kg, 1 µL) to verify possible nonspecific interaction by cannabinoid ligands. Both antagonists were dissolved in dimethyl sulfoxide 50% (DMSO).

The hypercapnic (7% CO_2_, 21% O_2_, balanced with N_2_) and hypoxic (10% O_2_, balanced with N_2_) gas mixture was purchased from White Martins Gases Industriais Ltd.a (Sertãozinho, SP, Brazil).

### 4.4. Determination of Pulmonary Ventilation

Measurements of pulmonary ventilation (${\dot{V}}_{E}$) were performed using the whole body plethysmography method, based on the technique described by Drorbaugh and Fenn [57] and used previously by our group [58,59,60]. In brief, freely-moving rats were kept in a 5-L chamber ventilated with room air or a hypercapnic gas mixture containing 7% CO_2_ (White Martins, Sertãozinho, Brazil) or a hypoxic gas mixture containing 10% O_2_ (White Martins). The flow rate of the inflow gas into the animal chamber was monitored by a flowmeter (model 822-13-OV1-PV2-V4, Sierra Instruments, Monterey, CA, USA). During the measurements, the flow was interrupted, and the chamber was sealed for approximately 2 min; the pressure oscillations due to ventilation were monitored by a differential pressure transducer (TSD 160A, Biopac Systems). The signals were fed into a pre-amplifier (DA 100C, Biopac Systems), passed through an analog-to-digital converter, and digitized on a microcomputer equipped with data acquisition software (MP100A-CE, Biopac Systems). The sampling frequency was 200 Hz. The results were analyzed using data analysis software (LabChart Software, version 7.3; ADInstruments, Sydney, Australia). Tidal volume (V_T_) and respiratory frequency (fR) were calculated to estimate ventilation per breath. V_T_ was calculated using an appropriate formula [57,61]. The calibration for volume was obtained during each experiment by injecting 1 mL of air into the animal chamber.

### 4.5. Blood Gases and pH Measurements

Blood gases, pH and bicarbonate were measured in the following groups: icv—DMSO (room air, hypercapnia and hypoxia), AMG9810 (room air, hypercapnia and hypoxia); ip—DMSO (room air and hypoxia), AMG9810 (room air and hypoxia). For pH, blood gases and bicarbonate measurements, two drops of blood were sampled for immediate analyses of arterial pH (pHa), arterial carbon dioxide partial pressure (*P*aCO_2_), arterial oxygen partial pressure (*P*aO_2_) and plasma bicarbonate (HCO_3_^–^). These samples were transferred to an EG7+ cartridge to be read in an i-STAT blood gas portable analyzer (i-STAT Analyzer, Abbott Laboratories, Libertyville Township, IL, USA). 

### 4.6. Experimental Protocol

Seven days after stereotaxic surgery, the animals were placed in a chamber (5 L) and Tb was continuously measured using the “dataloggers”. The chamber was initially ventilated with room air (21% O_2_) for a period of acclimatization of at least 30 min, and then two measurements of ventilation and MAP were performed and blood was collected for measurements of pH and blood gases. The animals were then given icv injection of the vehicle or TRPV1 channel antagonist (AMG9810 or AMG7905). After microinjection, the animals were maintained in normoxic normocapnia for 20 min. Cardiorespiratory measurements were performed at 2, 5, 10, 15, 20 min after microinjection. Twenty minutes after microinjection, the second set of measurements of blood gases and pH were performed. After 25 min of normoxic normocapnia, the chamber was ventilated with another gas mixture, hypercapnia (7% CO_2_, 21% O_2_, balanced with N_2_) or hypoxia (10% O_2_, equilibrated with N_2_) for another 35 min. Cardiorespiratory measurements were performed at 30, 35, 40, 45, 50 and 60 min after microinjection and blood gases and pH were measured at 60 min, at the end of the hypoxic or hypercapnic challenges. Finally, the rat was returned to normoxic normocapnia for 30 min to recover baseline ventilation and blood pressure. After returning to baseline, the last measurement was performed at 90 min. Hypoxia and hypercapnia were performed in different animals.

To verify if the effect of the AMG9810 was central or peripheral, another group of animals received intraperitoneal injection of the drug (2.85 µg/kg, 1 mL/kg). The same measurements for ${\dot{V}}_{E}$, HR, MAP and Tb were performed. Blood was collected at the same moment as central injection for blood gases and pH measurements.

### 4.7. Data Processing and Analysis

The results were reported as mean ± SEM. Cardiorespiratory variables were calculated using 2-min intervals of respiratory and pulsatile arterial pressure recordings when the animals were quiet and presenting no body movements. The respiratory, cardiovascular, thermal and blood gas variables were compared between vehicle and TRPV1 antagonist groups across the time points by two-way repeated measures ANOVA, followed by Tukey’s test used for multiple comparisons. A *p* value of <0.05 was considered to be statistically significant.

## Figures and Tables

**Table 1:** Values of arterial pH (pHa), arterial carbon dioxide partial pressure (PaCO_2_), arterial oxygen partial pressure (PaO_2_) and plasma bicarbonate (HCO_3_^–^) of Wistar rats in vehicle (DMSO)- and AMG9810 (2.85 µg/kg)-treated groups before microinjection (b.m.), at the end of 20 min of normocapnia or normoxia after microinjection (a.m.) and at the end of hypercapnia (**A**) or hypoxia (**B**) exposure after microinjection (a.m.).

**A**	**DMSO (n = 7)**			**AMG9810 (n = 7)**		
	**Normocapnia** **(b.m.)**	**Normocapnia** **(a.m.)**	**Hypercapnia** **(a.m.)**	**Normocapnia** **(b.m.)**	**Normocapnia** **(a.m.)**	**Hypercapnia** **(a.m.)**
pHa	7.44 ± 0.01	7.43 ± 0.01	7.30 ± 0.01 *	7.44 ± 0.01	7.43 ± 0.01	7.28 ± 0.01 *
*P*aCO_2_ (mmHg)	27.2 ± 1.4	26.4 ± 1.5	42.0 ± 1.8 *	26.8 ± 1.3	25.3 ± 1.4	38.2 ± 0.8 *
*P*aO_2_ (mmHg)	78.3 ± 1.9	80.1 ± 1.6	110.0 ± 1.3 *	76.0 ± 1.6	79.0 ± 1.4	110.3 ± 1.9 *
HCO_3_^−^	18.7 ± 1.5	17.6 ± 1.2	20.9 ± 1.1	18.2 ± 1.1	16.9 ± 0.9	18.0 ± 0.7
**B**	**DMSO (n = 7)**			**AMG9810 (n = 6)**		
	**Normoxia** **(b.m.)**	**Normoxia** **(a.m.)**	**Hypoxia** **(a.m.)**	**Normoxia** **(b.m.)**	**Normoxia** **(a.m.)**	**Hypoxia** **(a.m.)**
pHa	7.44 ± 0.01	7.44 ± 0.01	7.58 ± 0.01 **	7.46 ± 0.01	7.44 ± 0.01	7.58 ± 0.01 **
*P*aCO_2_ (mmHg)	29.3 ± 2.1	25.9 ± 0.8	14.8 ± 0.5 **	28.4 ± 1.4	26.1 ± 0.7	13.7 ± 0.5 **
*P*aO_2_ (mmHg)	69.0 ± 2.2	76.0 ± 1.5	28.7 ± 1.2 **	72.8 ± 2.1	78.8 ± 2.2	31.1 ± 1.3 **
HCO_3_^−^	20.3 ± 1.8	18.3 ± 0.8	14.1 ± 0.3 **	20.0 ± 0.9	17.8 ± 0.6	13.0 ± 0.4 **

* Means statistical difference between the time before microinjection and at the end of hypercapnia exposure after microinjection in the same group. ** Means statistical difference between the time before microinjection and at the end of hypoxia exposure after microinjection in the same group.

**Table 2:** Values of arterial pH (pHa), arterial carbon dioxide partial pressure (PaCO_2_), arterial oxygen partial pressure (PaO_2_) and plasma bicarbonate (HCO_3_^–^) of Wistar rats in vehicle (DMSO)- and AMG9810 (2.85 µg/kg)-treated groups before microinjection (b.m.), at the end of 20 min of normoxia after microinjection (a.m.) and at the end of hypoxia exposure after microinjection (a.m.).

	DMSO (n = 3)			AMG9810 (n = 4)		
	Normoxia (b.m.)	Normoxia (a.m.)	Hypoxia (a.m.)	Normoxia (b.m.)	Normoxia (a.m.)	Hypoxia (a.m.)
pHa	7.46 ± 0.01	7.47 ± 0.01	7.65 ± 0.02 *	7.43 ± 0.01	7.46 ± 0.01	7.60 ± 0.02 *
*P*aCO_2_ (mmHg)	38.6 ± 2.2	36.9 ± 2.1	18.6 ± 1.4 *	35.4 ± 2.7	36.7 ± 1.3	14.0 ± 1.1 *
*P*aO_2_ (mmHg)	75.6 ± 2.2	76.3 ± 0.8	30.1 ± 1.0 *	70.5 ± 3.4	79.0 ± 3.6	32.7 ± 1.2 *
HCO_3_^−^	27.8 ± 1.7	27.2 ± 1.2	17.6 ± 1.8 *	23.1 ± 2.2	28.9 ± 0.9	13.1 ± 0.6 *

* Means statistical difference between the time before microinjection and at the end of hypoxia exposure after microinjection in the same group.
